# Clinical significance of intermittently absent end-diastolic flow of the fetal umbilical artery on perinatal and neonatal outcomes

**DOI:** 10.1590/1806-9282.20242005

**Published:** 2025-07-07

**Authors:** Zeynep Kayaoglu Yildirim, Alperen Ince, Isil Turan Bakirci, Gokhan Bayanmelek, Gokhan Bolluk

**Affiliations:** 1 Başakşehir Çam ve Sakura City Hospital, Department of Perinatology – İstanbul, Turkey.; 2 Başakşehir Çam ve Sakura City Hospital, Department of Obstetrics and Gynecology – İstanbul, Turkey.

**Keywords:** Doppler ultrasonography, Intrauterine growth restriction, Neonatal health, Pregnancy outcome, Umbilical artery

## Abstract

**OBJECTIVE:**

The aim of this study was to compare the perinatal outcomes of pregnancies with intermittently absent end-diastolic umbilical artery Doppler flow and persistently absent flow, focusing on their implications for clinical management.

**METHODS:**

This retrospective cohort study was conducted at Basaksehir Cam and Sakura City Hospital between 2020 and 2023, and included 137 pregnancies diagnosed with either intermittently absent end-diastolic umbilical artery Doppler flow (n=38) or persistently absent flow (n=99). Demographic data, pregnancy complications, delivery details, and neonatal outcomes were analyzed.

**RESULTS:**

The mean latency from diagnosis to delivery was statistically significantly longer in the intermittently absent end-diastolic umbilical artery Doppler flow group (15.6 days) than in the persistently absent flow group (7.88 days; p<0.0001). The intermittently absent end-diastolic umbilical artery Doppler flow group demonstrated better outcomes, with a higher gestational age at delivery and improved Apgar scores. Notably, although the difference was not statistically significant, all stillbirths (n=4) occurred in the persistently absent flow group. No intergroup differences were found in terms of intensive care unit admission or neonatal intubation.

**CONCLUSION:**

This study highlights an important prognostic distinction between intermittently absent end-diastolic umbilical artery Doppler flow and persistently absent flow, with intermittently absent end-diastolic umbilical artery Doppler flow representing a less severe form of placental insufficiency. These findings provide valuable insights for optimizing antenatal surveillance strategies, suggesting that intermittently absent end-diastolic umbilical artery Doppler flow cases may be candidates for outpatient management, whereas persistently absent flow cases require more intensive monitoring.

## INTRODUCTION

Doppler velocity measurement of the umbilical artery (UA) serves as a crucial clinical tool for monitoring feto-placental hemodynamics and assessing fetal well-being in high-risk pregnancies^1,2^. This technique has demonstrated notable advantages in monitoring potentially risky fetuses, particularly in pregnancies complicated by placental disorders, such as fetal growth restriction (FGR)^3,4^. Since UA Doppler (UAD) results can deteriorate over the course of pregnancy, regular assessments are recommended^5,6^. However, the frequency of these assessments, management protocols, and delivery timing recommendations vary among major national obstetric societies^7^.

The UA blood velocity waveform is typically characterized by the pulsatility index (PI) and qualitative information concerning the potential absence or reversal of end-diastolic flow (EDF)^8^. Absent end-diastolic flow (AEDF) signifies increased resistance to flow in the placental vascular bed^9^ and is associated with elevated risks of intrauterine death and adverse perinatal outcomes^10^. AEDF in the UA can be either persistent (pAEDF), occurring in most or all fetal cardiac cycles, or intermittent (iAEDF), manifesting in only some of the cardiac cycles. However, there is a lack of standardized definitions for these terms, and the clinical implications of the iAEDF versus the pAEDF remain unclear.

A previous study indicated that, compared to fetuses with pAEDF, those with iAEDF are diagnosed with UAD abnormalities later in pregnancy and are delivered at a later gestational age (GA) with higher birth weights^11^. Consequently, it is plausible that some fetuses with an iAEDF may remain in utero for an extended duration without facing an immediate risk of death. Identifying this specific subgroup might allow the avoidance of some neonatal risks associated with extremely preterm birth.

We aimed to assess the risk of adverse perinatal outcomes among pregnant patients with iAEDF and pAEDF. This investigation seeks to contribute to a better understanding of the fetal risks associated with different patterns of AEDF, thereby informing clinical management and decision-making for high-risk pregnancies.

## METHODS

‘This is a retrospective cohort study of nonanomalous, singleton pregnancies diagnosed with either iAEDF or pAEDF. The study was approved by the Clinical Research Ethics Committee of Başakşehir Çam ve Sakura City Hospital (Ethics No: 2023-561, Date: 08/11/2023). The study is also consistent with the most recent version of the Declaration of Helsinki (2019/92). As this was a retrospective research, no informed consent was obtained from the participants. Patients with major fetal anomalies or aneuploidies, missing birth and/or neonatal outcome data, or those whose UAD studies demonstrated reversed end-diastolic flow (REDF) before an AEDF diagnosis, were excluded. Fetuses were separated into two groups: temporary empty EDF (iAEDF) and empty EDF without returning (pAEDF). It was confirmed from ultrasound and digital records that the patients were evaluated in accordance with the perinatology clinic follow-up and treatment protocol of Başakşehir Çam ve Sakura City Hospital. All patients underwent the abdominal ultrasound examination, and the free-floating loop of the umbilical cord was used to visualize the UAD waveforms. To improve the accuracy of the measurements, waveforms were obtained during a brief pause in maternal breathing and in the absence of fetal breathing movements. At least three separate UAD assessments were performed for each fetus. The UA PI was calculated, and patients with a UA PI above the 95th percentile for GA were included. Doppler waveforms were interpreted as iAEDF if the absence of diastolic flow was observed in one or more waveforms during at least one image cycle in all three assessments. The complete absence of diastolic flow in all waveforms was considered a persistent absence. REDF was interpreted as flow reversal in the fetal UA in one or more cardiac cycles. Patients defined as having iAEDF were not subdivided according to the percentage of patients without waveforms. The latest guidelines on the management of FGR from the Society for Maternal-Fetal Medicine and the American College of Obstetricians and Gynecologists (ACOG) published in 2020 recommend that pregnant patients with AEDF undergo UAD surveillance 2–3 times a week and deliver at 33–34 weeks of gestation ^5,12^. Inpatient treatment for AEDF is recommended as an option. In our study, AEDF management included admitting pregnant patients for antenatal corticosteroid administration and inpatient monitoring, including daily UAD assessment and twice-daily antenatal testing. Unless otherwise indicated, delivery was performed at 34 weeks, in accordance with ACOG recommendations^13^. Each case was individually evaluated using a multidisciplinary approach involving perinatology, neonatology, and anesthesiology teams to determine the optimal timing of delivery. Emergency delivery was performed before 34 weeks in the presence of maternal indications requiring urgent interventions, such as uncontrolled maternal hypertension, severe preeclampsia or eclampsia, Hemolysis, Elevated Liver enzymes, Low Platelet count (HELLP) syndrome, maternal sepsis or chorioamnionitis, and placental abruption, or in the presence of significant fetal hypoxia detected via nonstress test (NST) or biophysical profile (BPP). Additionally, if abnormal UAD findings progressed to REDF, delivery was scheduled at 32 weeks of gestation unless the absence of the a-wave on the fetal ductus venosus Doppler was detected. Demographic data, pregnancy complications, antenatal follow-up data, and delivery and neonatal outcomes were obtained from hospital digital records.

The main measure evaluated was a combination of various neonatal measurements such as birth weight, Z-score of birth weight at discharge, Apgar score at the first minute, Apgar score at the fifth minute, 5-min Apgar scoring of <7, the use of ventilatory support or surgical intensive care unit (ICU), and/or intubation. The secondary outcome measures were the review of patients’ demographic data, the time took to arrive at an AEDF diagnosis, the time duration from reaching the AEDF diagnosis to delivery, and complications of pregnancy (intrauterine fetal death [IUFD] ablatio placenta, fetal distress, intrauterine growth restriction [IUGR], and amniotic fluid abnormalities).

These outcomes were assessed as inter-group differences in patients with iAEDF versus patients with pAEDF.

### Statistical analysis

Statistical analysis was performed using R statistical software (R Core Team 2021). p<0.05 was considered statistically significant. The normality of variable distributions was assessed using quantile–quantile plots and the Shapiro-Wilk test. For continuous variables, the unpaired Student's t-test or Mann-Whitney U test was used, depending on the normality of the distribution. Categorical variables were analyzed using the χ^2^ test or Fisher's exact test, depending on variable counts^14^.

## RESULTS

Our cohort included 137 pregnancies in the final analysis. A total of 99 (33.0%) patients were classified as pAEDF, while 38 (16.0%) were classified as iAEDF.

Table 1 represents results from the comparison of the two groups in terms pregnancy outcomes including the week of AEDF diagnosis, gestational age at birth, birthweight, z score for standardized birthweight and the time passed from the diagnosis to the birth. Mean gestational age at birth in women who diagnosed with pAEDF was 29.6±2.8 while mean gestational age at birth in women who diagnosed with iAEDF 30.9±2.6 (p=0.03). The time passed from the diagnosis to the birth was much shorter for women who diagnosed with pAEDF (mean=7.88, sd=9.99) than for women who diagnosed with iAEDF (mean=15.6, sd=11.5) (p≤0.0001).

**Table 1:** Pregnancy outcomes.

	iAEDF (n=38)	pAEDF (n=99)	p-value
The week of AEDF diagnosis	28.6 (2.8)	28.5 (2.9)	0.84^a^
Gestational age at birth	30.9 (2.6)	29.6 (2.8)	0.03*
Birthweight	1067 (450)	929 (441)	0.09*
z score for standardized birthweight	(-3.05) (1.1)	(-3.0) (1.6)	0.73*
The time passed from the diagnosis to the birth	15.6 (11.5)	7.88 (9.99)	<0.0001*

* The Mann-Whitney U,

a Independent Sample T-test.

Table 2 represents results from the comparison of the two groups in terms pregnancy complications including AFV abnormalities (A: anhydramnios O: oligohydramnios, P: polyhydramnios), IUFD (intrauterine fetal demise), placental ablation, fetal distress. There were no statistically significant differences between two groups in terms of pregnancy complications. However, the percentage of fetal distress in women who diagnosed with pAEDF (56.6%) was noticeably greater than the percentage of fetal distress in women who were diagnosed with intermittent dak (39.5%). Although there was no significant difference between the groups in terms of stillbirths, all four stillborns were in the pAEDF group.

**Table 2:** Pregnancy complications.

		iAEDF (n=38)	pAEDF (n=99)	p-value
AFV				
	A	4 (10.5)	9 (9.1)	0.57**
	N	29 (76.3)	66 (66.7)	
	O	5 (13.2)	21 (21.2)	
	P	0 (0.0)	3 (3.0)	
IUFD				
	+	0 (0.0)	4 (4.0)	0.57**
	-	38 (100.0)	95 (96.0)	
Placental ablation				
	+	1 (2.6)	5 (5.1)	1.00**
	-	37 (97.4)	94 (94.9)	
Fetal distress				
	+	15 (39.5)	56 (56.6)	0.07***
	-	23 (60.5)	43 (43.4)	
IUGR				
	+	36 (94.7)	93 (93.9)	1.00**
	-	2 (5.3)	6 (6.1)	

** Fisher's Exact test,

*** Chi-square test.

The comparison of the two groups in terms adverse neoatal outcomes is given in Table 3. Among women who were in the pAEDF group, mean Apgar score in the first minute was 4.2±2.2, while the mean Apgar score in the first minute among women who were in the iAEDF group was 5.4±1.9 (p=0.004). Similarly, the mean Apgar score in the fifth minute was lower for the pAEDF group than the iAEDF group (6.4±2.2, 7.6±1.0 respectively).

**Table 3:** Adverse neonatal outcomes.

		iAEDF (n=38)	pAEDF (n=99)	p-value
Apgar score in the first minute		5.4 (1.9)	4.2 (2.2)	0.004*
Apgar score in the fifth minute		7.6 (1.0)	6.4 (2.2)	0.002*
Apgar score in the fifth minute				
	<7	5 (13.2)	33 (33.3)	0.02***
	³7	33 (86.8)	66 (66.7)	
Admission to the ICU				
	+	0 (0.0)	4 (4.0)	0.58**
	-	38 (100.0)	95 (96.0)	
To be intubated				
	+	20 (52.6)	68 (68.7)	0.08***
	-	18 (47.4)	31 (31.3)	

* The Mann-Whitney U,

** Fisher's Exact test,

*** Chi-square test.

The percentage of women with Apgar score in the fifth minute less than 7 in the dak group (33.3%) was much higher than the percentage the percentage of women with Apgar score in the fifth minute less than 7 in the intermittent dak group (13.2%) (p=0.02).

The significant difference between the two groups in terms of Apgar score was due to the higher mean GA at birth of women diagnosed with pAEDF compared to the mean GA at birth of women diagnosed with iAEDF.

There were no significant differences between the two groups in terms of admission to the ICU or intubation.

## DISCUSSION

In the current study conducted on pregnancies with AEDV, pregnancies with iAEDF had longer latency from diagnosis to delivery, were delivered at a later GA, and were less likely to be delivered because of fetal distress. Based on the data that pregnant women with iAEDF have a longer latency from diagnosis to delivery and require delivery later in gestation, iAEDF is a stage of placental insufficiency progression and likely represents less severe UAD abnormality than pAEDF.

Rosner et al. compared outcomes among 109 pregnancies with an iAEDF or pA/REDF from 19 to 39 weeks^10^. Similar to our study, they reported that pregnancies with an iAEDF were delivered at a later GA and were less likely to have a fetal indication for delivery. In contrast, they found no difference in latency from AEDV diagnosis to delivery and did not observe changes in UAD velocimetry. This may be attributed to the low percentage of pregnancies complicated by FGR in this study. Their study included all pregnancies with UAD abnormalities, as did ours, and only 83% of the subjects had FGR. In our study, 94% of the patients had UAD abnormalities complicated with FGR.

Similar to our study, a recent retrospective study of singletons with FGR and absent end-diastolic velocity conducted by Bligard et al. has reported that the latency to delivery was longer in the iAEDF group than in the pAEDF group and the nonreassuring fetal status indication for delivery was greater in the pAEDF group. In their study, when the two groups were compared in terms of Apgar scores and birth weights, the Apgar scores and birth weights were greater for fetuses complicated with iAEDF^15^. Our study supported these data with approximately twice the number of patients.

Green et al. compared outcomes among three Doppler groups (persistently elevated, intermittently absent, and persistently AEDF) of growth-restricted fetuses^16^. According to our study, there was no difference in composite neonatal morbidity among the three groups. Although the Apgar score was lower in the pAEDF group, there were no significant differences between the two groups in terms of admission to the ICU or intubation. Our study is limited because subsequent neonatal outcomes are unknown. It is important to acknowledge that UAD is a dynamic measurement that changes over time. Green et al.'s study and our study could have been more powerful if they had included data from pregnancies with an iAEDF that subsequently progressed to an AEDF in the outcome analyses.

## CONCLUSION

In fetuses with AEDF in the UA, the severity of flow absence was associated with pregnancy outcomes (GA at birth, time elapsed from diagnosis to birth, fetal distress, and Apgar scores). Although there was no significant difference between the groups in terms of stillbirth, the fact that all four stillborns are in the pAEDF group suggest that the inpatient follow-up of pregnancies with iAEDF allows early intervention in these patients. However, the mean time from diagnosis to delivery in women with an iAEDF was 15.6 days, an extended latency from diagnosis to delivery, which may favor the outpatient management of iAEDF. However, considering the lack of available clinical data on the iAEDF population, we believe that our study contributes to the current data as a descriptive study because it involves the largest number of patients with AEDF and the multiple parameters evaluated.

## Data Availability

The datasets generated and/or analyzed during the current study are available from the corresponding author upon reasonable request.
